# Nutritional values of wild edible freshwater macrophytes

**DOI:** 10.7717/peerj.15496

**Published:** 2023-07-11

**Authors:** Muta Harah Zakaria, Shiamala Devi Ramaiya, Nordiah Bidin, Nurul Nur Farahin Syed, Japar Sidik Bujang

**Affiliations:** 1 Department of Aquaculture, Faculty of Agriculture, Universiti Putra Malaysia, UPM Serdang, Selangor Darul Ehsan, Malaysia; 2 Universiti Putra Malaysia (UPM), International Institute of Aquaculture and Aquatic Sciences (I-AQUAS), Port Dickson, Negeri Sembilan, Malaysia; 3 Department of Crop Science, Faculty of Agriculture Sciences and Forestry, Universiti Putra Malaysia Bintulu Campus, Bintulu, Sarawak, Malaysia; 4 Department of Biology, Faculty of Science, Universiti Putra Malaysia, UPM Serdang, Selangor Darul Ehsan, Malaysia

**Keywords:** Freshwater macrophytes, Nutrients, Proximate analysis, Wild plants, Mineral content

## Abstract

**Background:**

The social acceptability of wild freshwater macrophytes as locally consumed vegetables is widespread. Freshwater macrophytes have several uses; for example, they can be used as food for humans. This study determined the proximate composition and mineral content of three freshwater macrophyte species, *i.e.*, *Eichhornia crassipes*, *Limnocharis flava*, and *Neptunia oleracea*.

**Methods:**

Young shoots of *E. crassipes*, *L. flava*, and *N. oleracea* were collected from shallow channels of Puchong (3°00′11.89″N, 101°42′43.12″E), Ladang 10, Universiti Putra Malaysia (2°58′44.41″N, 101°42′44.45″E), and Kampung Alur Selibong, Langgar (06°5′50.9″N, 100°26′49.8″E), Kedah, Peninsular Malaysia. The nutritional values of these macrophytes were analysed by using a standard protocol from the Association of Official Analytical Chemists. Eight replicates of *E. crassipes* and *L. flava* and four replicates of *N. oleracea* were used for the subsequent analyses.

**Results:**

In the proximate analysis, *N. oleracea* possessed the highest percentage of crude protein (29.61%) and energy content (4,269.65 cal g^−1^), whereas *L. flava* had the highest percentage of crude fat (5.75%) and ash (18.31%). The proximate composition trend for each species was different; specifically, all of the species possessed more carbohydrates and fewer crude lipids. All of the species demonstrated a similar mineral trend, with high nitrogen and potassium and lower copper contents. Nitrogen and potassium levels ranged from 12,380–40,380 mg kg^−1^ and from 11,212-33,276 mg kg^−1^, respectively, and copper levels ranged from 16–27 mg kg^−1^. The results showed that all three plant species, i.e., *E. crassipes, N. oleracea*, and *L. flava* are plant-based sources of macro- and micronutrient beneficial supplements for human consumption.

## Introduction

One of the top ten factors contributing to mortality was the low intake of vegetables and fruits (Ezzati et al., 2002). Vegetables are sources of vitamins and minerals for the antioxidant activity that is needed in the diet to meet the daily micronutrient requirements (Gupta & Bains, 2006). To reduce individual risk and cardiovascular disease, humans and animals require optimal intakes of minerals such as potassium, sodium, calcium, magnesium, copper, manganese, iodine, and zinc (Mertz, 1982). To perform physiological functions, micronutrients such as copper, zinc, and iron obtained from food are required in the human body in limited amounts (typically less than 100 micrograms per day). Micronutrient deficiency (*e.g.*, zinc deficiency) causes decreased taste acuity, slow wound healing, impaired development, decreased sexual maturity, impaired immune system function, and impaired metabolism and homeostasis disorders of the thyroid gland (Almatsier, 2006).

Freshwater macrophytes are aquatic plants submerged, emerging, or floating on the water surface (Lacoul & Freedman, 2006). They occur in swamps, peatlands, lakes, streams, ponds, rice fields, and drainage canals (Den Hartog, 1981; Muta Harah et al., 2005). Wild plants have played a crucial role in the human diet, and some communities still depend on these wild foods (Tbatou et al., 2018). According to Grubben, Siemonsma & Kasem (1994), of the 225 vegetables in Southeast Asia, approximately 100 species are wild weeds. In East Malaysia (*i.e.,* Sarawak), some 43–48 species of wild freshwater macrophytes belonging to 28 families that are considered as weeds are collected and used as edible food and food preparation, medicine, household items for pillows and mats, and even used to make souvenirs (Muta Harah et al., 2005; Muta Harah, Japar Sidik & Suzalina Akma, 2014). The local collectors also offer freshwater macrophytes for sale in the local markets. Wild freshwater macrophytes are slowly being well received as consumed vegetables (Muta Harah et al., 2005; Saupi, Zakaria & Bujang, 2009; Muta Harah, Japar Sidik & Suzalina Akma, 2014; Noorasmah et al., 2015; Noorasmah et al., 2016). In addition to being cheaper, vegetarian products are also important as sources of essential minerals in human nutrition (Saupi, Zakaria & Bujang, 2009; Noorasmah et al., 2015; Caunii et al., 2010). Some indigenous leafy vegetables, nuts, and wild fruits also provide energy and are food supplements with good levels of carbohydrates and other nutrients (Achinewhu, Ogbonna & Hart, 1995).

Global food problems have challenged all organizations and researchers to investigate the possibility of using wild plant species as supplementary sources of nutrients (Abubakar et al., 2021). Wild plants can provide minerals, vitamins, proteins, phenolics, carotenoids, and carbohydrates (Seal, Pillai & Chaudhuri, 2017; Ghanimi et al., 2022). Studies on the nutritional potential of some wild edible plants have demonstrated their comparability or even superiority to domesticated crops (Shad, Shah & Bakht, 2013). Global dietary guidelines recommend increased consumption of fruits and vegetables to mitigate the threat of diet-related diseases, including metabolic disorders, cancer, and cardiovascular diseases (Stratton et al., 2021). Therefore, the promotion of these plants will ensure important nutritional sources for food security and sustainable development.

**Figure 1:**
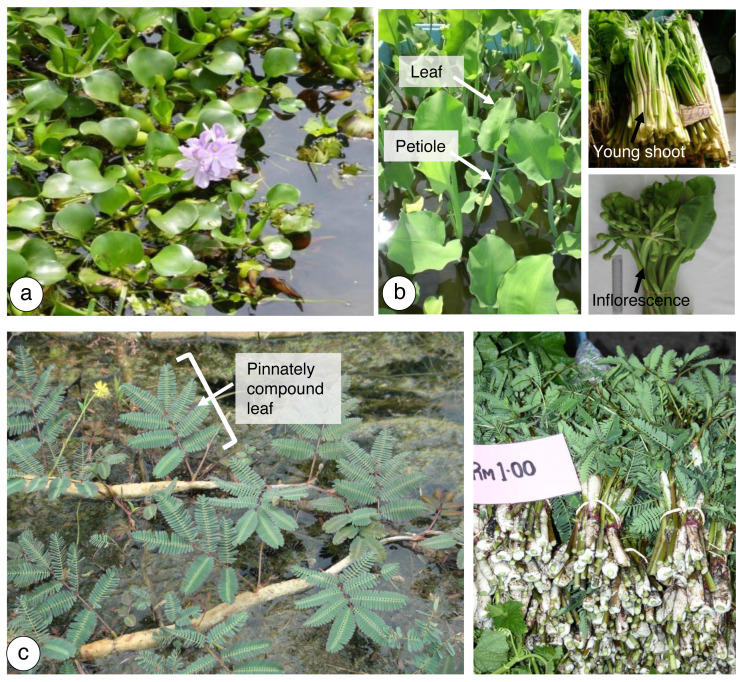
Freshwater macrophytes in study sites. (A) *E. crassipes* from a shallow channel of Puchong, Selangor, Malaysia, (B) *L. flava* from a shallow channel of Ladang 10, Universiti Putra Malaysia, Selangor, Malaysia. Young shoots and inflorescences of (A) and (B) are consumed cooked as vegetables, (C) *N. oleracea* from a shallow channel of Kg. Alur Selibong, Langgar, Kedah. The young leaves’ shoot tips are usually consumed blanched or cooked as a vegetable.

Macrophytes have been used locally in Malaysia as food sources (Saupi, Zakaria & Bujang, 2009; Muta Harah, Japar Sidik & Suzalina Akma, 2014; Noorasmah et al., 2015; Noorasmah et al., 2016). The common macrophyte species that are highly ingested by local residents include *Eichhornia crassipes* (Mart.) Solms, *Limnocharis flava* (L.) Buchenau, and *Neptunia oleracea* Lour. (Fig. 1). *Eichhornia crassipes*, which is known as water hyacinth, is a floating freshwater macrophyte with broad leaves above the water surface and spongy stalks (Fig. 1A). *Limnocharis flava* (yellow velvetleaf) is a plant with pale green leaves and stalks with triangular petiole leaves (Fig. 1B). The leaf blade is papery and broadly ovate-elliptic. Moreover, water mimosa, which is known as *N. oleracea*, has sensitive leaves when touched. It has white spongy air tissues on stems floating on the surface of freshwater (Fig. 1C). Under uncontrolled conditions, these species cause problems in human-made water bodies. Due to their fast rate of reproduction in vegetative and generative states, these plants are commonly referred to as noxious weeds. Rather than destroying them with herbicides, which may affect the ecology, it is preferable to collect and consume them or to use them as for feed.

Local people have been harvesting the young leaves with petioles and inflorescences of *E. crassipes* and *L. flava*, young leaf shoot tips, and immature pods of *N. oleracea* to be consumed as blanched or cooked vegetables, as well as to be sold in local markets to earn income. This scenario is also supported by studies on ethnobotanical information (Saupi, Zakaria & Bujang, 2009; Muta Harah, Japar Sidik & Suzalina Akma, 2014; Saupi et al., 2020) that investigated the most consumed wild aquatic plant species, particularly in the Bintulu and Sarawak communities. Based on the data, young shoots of *L. flava* and *N. oleracea* were commonly used in dish preparations due to their good palatability and sweet taste with great nutritional quality; however, these species are the least frequently available in the market. Due to the fact that the chemical compositions of aquatic macrophytes vary greatly depending on their species, seasons, habitat, and geographic location, proximate analyses and mineral studies are crucial in determining their nutritional value for future usage possibilities. In addition, a lack of documentation has been published on the nutritional profiling of these macrophytes. Therefore, the present study aimed to determine the proximate composition and mineral content of the commonly consumed wild freshwater macrophytes of *E. crassipes, L. flava*, and *N. oleracea*. The findings of this study may also suggest that these species should be commercially grown as new vegetable crops.

## Materials & Methods

### Sample collection and preparation

Plants of *E. crassipes*, *L. flava*, and *N. oleracea* were collected from shallow channels of Puchong (3°00′11.89″N, 101° 42′43.12 ″E), Ladang 10, Universiti Putra Malaysia (2°58′44.41 ″N, 101°42′44.45″E), and Kampung Alur Selibong, Langgar (06°5′50.9″N, 100°26′49.8″E), Kedah, Peninsular Malaysia, from January to March 2020. The complete specimen of each species was directly arranged on the drawing block, labelled, and pressed as soon as possible by using wooden presses and ensuring that the straps were tight enough to bind the press together. The herbarium samples were examined to verify the identity of the specific plant that was used in a study. The morphological characterization of the aquatic macrophytes was performed following the guidelines based on Soerjani, Kostermans & Tjitrosoepomo (1987).

Young shoots (light green with the tender shoot) were sampled and stored in a zip-lock plastic bag accordingly before being kept in an ice chest for transportation to the laboratory. Any adhering materials were removed from the plant samples by washing them with distilled water. Approximately 500 g of fresh samples were cut into small pieces and oven-dried at 60 °C until they reached a constant weight. Dry samples were ground by using an IKA A11^®^ Basic Analytical Mill (Ika, Stauffen, Germany) and passed through a 0.2 mm laboratory sieve. The sample powder was labelled and kept inside of an airtight container at room temperature prior to proximate composition (ash, moisture, crude lipid, crude protein, crude fibre, and energy) analysis and mineral content (nitrogen (N), phosphorus (P), potassium (K), calcium (Ca), magnesium (Mg), copper (Cu), manganese (Mn), zinc (Zn), and iron (Fe) analysis). Eight replicates (petiole and leaves) of *E. crassipes* and *L. flava* and four replicates of the whole pinnately compound leaf of *N. oleracea* were used for the subsequent analyses.

### Proximate composition analysis of the wild edible freshwater macrophytes

Proximate analyses of crude fibre composition, crude lipid, crude protein, ash, and moisture amount for the freshwater macrophytes were identified through the standard approaches of the AOAC (2000). The young shoot moisture was identified by placing the weighed fresh samples in an oven overnight or until a fixed weight was obtained at 60 °C, and the dried mass was determined. For ash determination, the initial crucible weight, which was already labelled and oven-dried (105 °C for 30 min), was measured. Samples (2 g) were put into the crucible and oxidized inside of a muffle furnace at 600 °C for 6 h. Afterwards, the samples were cooled overnight before being placed into the desiccator for 15 min and weighed until a constant weight was achieved. The ash amount was calculated following method 930.05.

Crude protein content was identified by placing 0.2 g of samples inside of a digestion tube and mixed with one tablet of Kjeltec Cu, 5 g K2SO4 + 0.5 g CuSO4, H2O, and 6 ml of concentrated sulfuric acid. The tube was inserted into the Turbotherm Digestor (Gerhardt, Germany) inside of a fume hood and digested for 2 h. The tube was left to cool for 30 min before being inserted into the Protein Analyser (Foss Tecator 2300 Kjeltec Analyser Unit; Foss Analytics, Hillerød, Denmark). The protein concentration was calculated as the percentage of nitrogen by using a conversion factor of 6.25 following method 955.04.

Petroleum ether from the samples was used to obtain crude lipids. Crude lipid was identified by using the 2055 Soxtec Avanti Manual System, Sweden (method 920.39), whereas crude fibre was calculated *via* acid–base digestion according to method 993.19. The estimation of the present carbohydrate level was performed regarding the difference by subtracting the overall percentage of crude protein, crude lipid, crude fibre, ash, and moisture from the 100% dry weight (DW) basis.

### Mineral content analysis of the wild edible freshwater macrophytes

The mineral contents of five macronutrients, including nitrogen (N), potassium (K), phosphorus (P), calcium (Ca), and magnesium (Mg), and four micronutrients, including zinc (Zn), iron (Fe), copper (Cu), and manganese (Mn), were analysed following the Association of Official Analytical Chemists (AOAC, 2000) method. All of the nutrient contents were determined by using AAS (Perkin Elmer 200 Flame Atomic Absorption Spectrophotometer, Waltham, MA, United States). The dried samples were milled to less than one mm in diameter. Additionally, the digestion tube was filled with 0.25 g of sample, and 5 ml of sulfuric acid (H_2_SO_4_) was then added. The tube was rotated until all of the plant material was moistened. The mixture was allowed to stand for at least 2 h. Afterwards, two mL of 30–35% hydrogen peroxide (H_2_O_2_) was added. Subsequently, the tube was placed into a port in a digestion block for 45 min at 285 °C. After 45 min, the tube was removed from the block and cooled for 10 min. H_2_O_2_ (two mL) was added if the sample was cloudy, and this process was repeated until the samples were transparent. The samples were then placed into a volumetric flask, filled with distilled water until a level of 100 mL was reached, and mixed. The sample solution was subsequently transferred into 100 mL plastic vials as a stock solution before analysis *via* AAS.

### Statistical analysis

**Figure 2:**
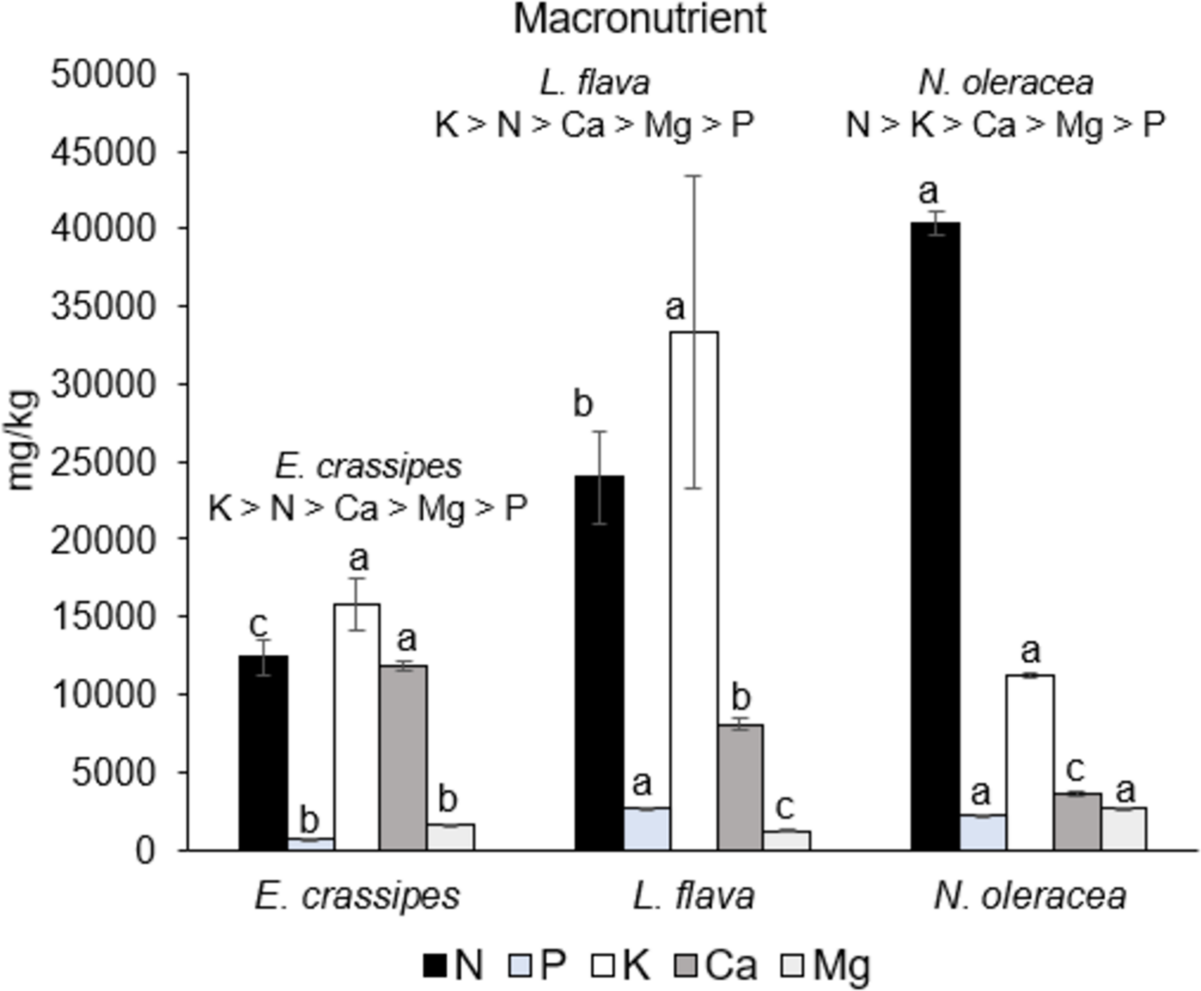
Macronutrient content in the three wild edible freshwater macrophytes.

**Figure 3:**
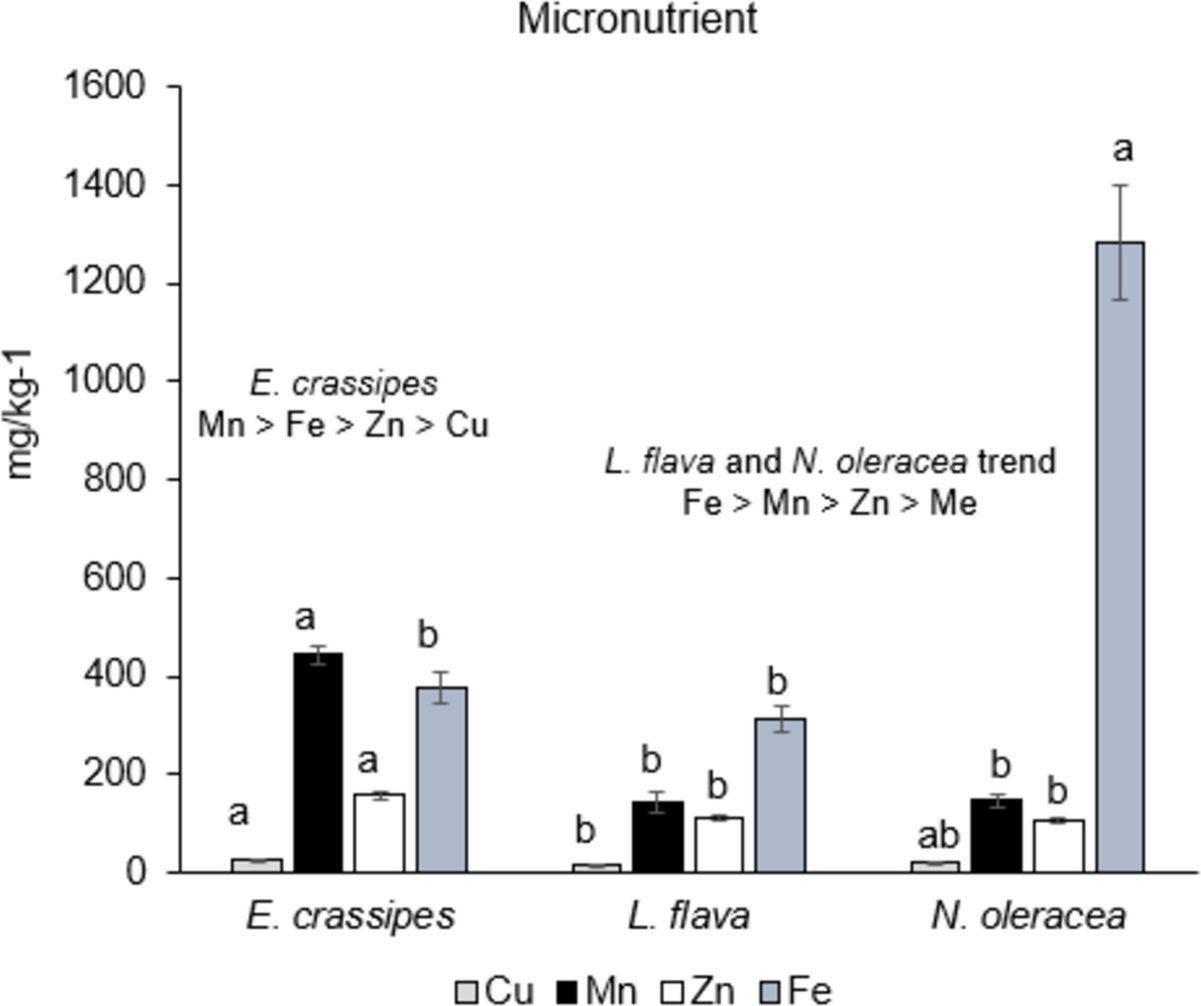
Micronutrient content in the three wild edible freshwater macrophytes.

The results are reported as the mean ± standard error. The data for proximate composition (Table 1) and mineral content (Figs. 2, 3) were statistically analysed by using SPSS, Statistical Software Program (IBM corporation, Armonk, NY, USA). Means were compared by using single-factor analysis of variance (ANOVA). Post-hoc Duncan’s Multiple Range Test (DMRT, *p* < 0.05) (Zar, 1999) was performed if the ANOVA result was significant. In addition, a multiple correlation analysis was performed to determine relationships between the abovementioned variables and freshwater macrophyte species. Principal component analysis (PCA) based on the Bray–Curtis similarity index was statistically analysed by using XLSTAT version 2014 software (Addinsoft, 2015, New York, USA) to obtain the relationship between the proximate composition and mineral content of freshwater macrophyte species in this study and for available data for other edible indigenous species.

**Table 1:** Proximate composition of freshwater macrophyte species and compares proximate composition (given as mean) of freshwater macrophyte species and other edible plant species.

**No.**	**Species**	**Moisture (%)**	**Ash (%)**	**Crude lipid (%)**	**Crude fibre (%)**	**Crude protein (%)**	**Carbohydrate (%)**	**Energy** **(cal g** ^−1^ **)**	**Trend**
**Freshwater macrophytes**									
1	*Eichhornia crassipes*	10.34 ± 0.76a (7.90–12.70)	13.23 ± 0.30b (11.93–14.14)	1.43 ± 0.26c (0.56–2.39)	21.34 ± 0.33a (20.16–22.58)	9.58 ± 0.73c (7.53–11.71)	54.42 ± 0.42a (52.89–56.05)	3395.15 ± 26.61b (3253.20–3481.90)	C>F>A>M>P>L
2	*Limnocharis flava*	7.99 ± 0.46b (6.60–9.70)	18.31 ± 0.92a (15.70–20.83)	5.75 ± 0.84a (2.62–8.07)	15.33 ± 1.18b (11.48–19.50)	16.58 ± 2.01b (11.21–22.09)	44.03 ± 0.91b (40.62–48.29)	3486.98 ± 151.34b (2996.10–3905.70)	C>A>P>F>M>L
3	*Neptunia oleracea*	10.82 ± 0.51a (9.33–11.50)	7.42 ± 0.04c (7.31–7.51)	3.48 ± 0.12b (3.13–3.64)	8.73 ± 0.30c (8.04–9.39)	29.61 ± 0.11a (29.32–29.87)	50.76 ± 0.29a (49.95–51.20)	4269.65 ± 31.08a (4203.80–4353.10)	C>P>M>F>A>L

df	2	2	2	2	2	2	2
F-value	5.572	52.334	14.480	44.218	35.564	103.928	14.216
P-value	0.014	5.4305E−8	0.000213	1.8342E−7	8.4202E−7	2.9351E−10	0.000235

**No.**	**Species**	**Moisture (%)**	**Ash (%)**	**Crude lipid (%)**	**Crude fiber (%)**	**Crude protein (%)**	**Carbohydrate (%)**	**Reference (s)**
4	*Limnocharis flava*	79.34	0.79	1.22	3.81	0.28	14.56	Saupi, Zakaria & Bujang (2009)
5	*Neptunia oleracea*	86.26	1.05	0.25	2.30	3.23	6.91	Noorasmah et al. (2015)
6	*Ipomoea aquatica*	72.83	10.83	11.00	17.67	6.30	54.20	Umar et al. (2007)
7	*Ipomoea aquatica*	51.36	2.75	0.81	1.20	1.70	42.18	Igwenyi et al. (2011)
**Vegetables**								
8	*Momordica balsamina*	71.00	18.00	2.66	29.00	11.29	39.05	Hassan & Umar (2006)
9	*Amaranthus hybridus*	59.30	4.10	1.20	3.40	4.60	27.40	Mepba, Eboh & Banigo (2007)
10	*Lycopersicon esculentum*	56.40	3.00	1.60	2.60	3.20	33.20	Mepba, Eboh & Banigo (2007)
11	*Telfaria occidentalis*	58.70	3.10	1.40	2.60	3.20	32.00	Mepba, Eboh & Banigo (2007)
12	*Basella alba*	54.80	3.10	0.90	2.70	4.20	34.30	Mepba, Eboh & Banigo (2007)
13	*Myrianthus arboreus*	83.90	16.40	13.10	11.60	18.75	40.15	Amata (2010)
14	*Moringa oleifera*	76.53	7.13	2.23	19.25	27.51	43.88	Oduro, Ellis & Owusu (2008)
**Spices**								
15	*Zingiber officinalis*	16.10	9.60	12.10	4.50	14.90	53.10	Achinewhu, Ogbonna & Hart (1995)
16	*Piper quineense*	55.30	4.50	11.70	8.70	26.60	38.90	Achinewhu, Ogbonna & Hart (1995)
**Medicinal plants**								
17	*Fagonia cretica*	9.30	16.00	10.40	25.10	7.70	31.50	Dastagir et al. (2013)
18	*Tribulus terrestris*	8.70	15.70	9.90	15.50	13.10	37.20	Dastagir et al. (2013)
19	*Chrozophora tinctoria*	9.70	16.00	13.00	6.70	10.50	44.10	Dastagir et al. (2013)
20	*Ricinus communis*	9.80	16.20	12.90	23.80	16.20	21.10	Dastagir et al. (2013)

**Notes.**

The varying superscript alphabets in the same column demonstrate the contrasts at *p* < 0.05 (ANOVA, Duncan’s Multiple Range Test test). Values are given as mean ± SE and range in parenthesis.

## Results

### Proximate compositions of edible freshwater macrophytes

The proximate composition of freshwater macrophytes is shown in Table 1. *Limnocharis flava* had significantly higher ash and crude lipid contents of 18.31 ± 0.92% and 5.75 ± 0.84%, respectively. In contrast, *N. oleracea* had a significantly higher crude protein of 29.61 ± 0.11% and an energy value of 4,269.65 ± 31.08 cal g^−1^, whereas the crude fibre content was observed to be high in *E. crassipes* (21.34%). The moisture and carbohydrate contents were comparable between *E. crassipes* and *N. oleracea,* ranging from 10.34–10.82% and 50.76–54.42%, respectively. Generally, carbohydrates had a higher concentration in all of the species, along with lower crude lipids. *Eichhornia crassipes* was categorically represented as carbohydrate >crude fibre >ash >moisture >protein >lipid, and this trend was contradictory to that of *L. flava* (carbohydrate >ash >protein >fibre >moisture >lipid) and *N. oleracea* (carbohydrate >protein >moisture >fibre >ash >lipid).

### The mineral content of edible freshwater macrophytes

Figure 2 shows the macronutrient content in the mineral analysis of the three edible freshwater macrophyte species. All of the macronutrient contents were significantly different (*p* < 0.05) between species(except for the K content). *Neptunia oleracea* had the highest N content (40,380.0 ± 730.6 mg kg^−1^), followed by *L. flava* (23,970.0 ± 3,022.0 mg kg^−1^) and *E. crassipes* (12,380.0 ± 1,129.9 mg kg^−1^). *Eichhornia crassipes* had the highest Ca content (11,863.5 ± 316.4 mg kg^−1^), whereas higher Mg values were demonstrated by *N. oleracea* at 2,616.0 ± 68 mg kg^−1^. The trend of macronutrient content shows that *E. crassipes* and *L. flava* are high in K >N >Ca >Mg >P, and *N. oleracea* is high in N >K >Ca >Mg >P.

The micronutrients were significantly different (*p* < 0.05) between the species. *Eichhornia crassipes* had the highest Cu, Mn, and Zn contents, whereas *N. oleracea* had a higher Fe content, as shown in Fig. 3. The trend of macronutrient content shows that *L. flava* and *N. oleracea* have similar trends with Fe >Mn >Zn >Cu, whereas the *E. crassipes* trend is Mn >Fe >Zn >Cu.

## Discussion

### Proximate composition and comparative analysis with previous studies

**Figure 4:**
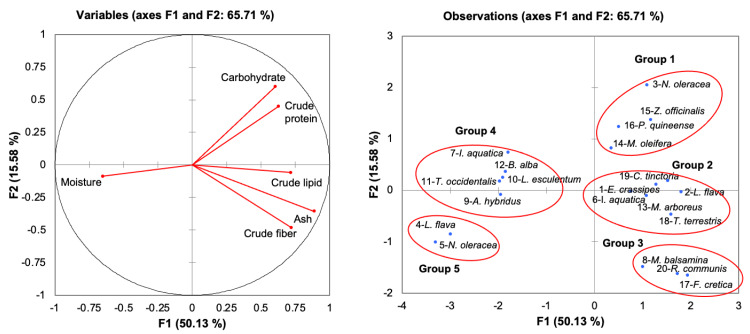
Principal component analysis of freshwater macrophytes with other edible plant species based on their proximate composition. (A) Plot of proximate composition (B) position of PC (principle component) score of species tested with other edible plants according to PC1 and PC2. No. 1–20 represents the assigned number of edible plant species as in Table 1 and corresponds to Groups 1, 2, 3, 4, and 5.

Table 1 shows the proximate composition of freshwater macrophytes, and other edible plants listed as vegetables, spices, and medicinal plants that are commonly consumed. The reference species were selected to examine the variation among the freshwater macrophytes in this study and previous studies (no 4–7), as well as among commonly consumed species. The objective of comparing freshwater macrophytes and other commonly consumed species is to demonstrate the potential of aquatic macrophytes as an alternative food to be consumed in daily life. The proximate composition in Table 1 was ordinated with a principal component analysis (PCA). The results in Fig. 4 (based on the Bray–Curtis similarity index at 50% similarity) showed that the total variance of the first two components was 65.71% (PC1 at 50.13% and PC2 at 15.58%). The low value of variance explained by F2 in the PCA is due to the separation of the plants based on their moisture content as defined by their positioning along F1, which represented more than 50% of the total variance of the analyses. The correlation matrix (Table 2) demonstrated a significant moderate correlation between ash and crude lipid (*r* = 0.616) and a strong correlation between ash and crude fibre (*r* = 0.767). In contrast, a moderate negative correlation (*r* =  − 0.525) was detected between ash and moisture.

There were five distinct groups (1, 2, 3, 4, and 5) displayed in the PCA (Fig. 4). Group 1 consisted of *N. oleracea* in the present study and clustered together with spice plants (*Z. officinali* s and *Piper guineense*) and *Moringa oleifera*, due to higher carbohydrate and protein contents. The current study grouped *E. crassipes* and *L. flava* with *I. aquatica, Myrianthus arboreus*, *Tribulus terrestris,* and *Chrozophora tinctoria* in Group 2 due to the comparable values of ash and crude lipids. Group 3 had plant species with higher fibre contents, including *M. balsamina, Fagonia cretica,* and *Ricinus communis*. In contrast, Group 5 consisted of freshwater macrophytes (*L. flava,* and *N. oleracea*), which had higher moisture content. The remaining species were grouped in Group 4 in the positive part of F1 with lower crude protein and carbohydrate contents.

**Table 2:** Correlation matrix for all variables in freshwater macrophyte species with other edible plant species.

**Variables**	**Moisture**	**Ash**	**Crude lipid**	**Crude fiber**	**Crude protein**	**Carbohydrate**
Moisture	1.000	−0.525^*^	−0.378	−0.269	−0.249	−0.366
Ash		1.000	0.616^*^	0.767^*^	0.339	0.381
Crude lipid			1.000	0.322	0.399	0.273
Crude fibre				1.000	0.347	0.237
Crude protein					1.000	0.431
Carbohydrate						1.000

**Notes.**

* Significant at *p* < 0.05.

The moisture content ranged from 7.99–10.82% (Table 1) of freshwater macrophytes in this study, which was much lower than the moisture content of 65.02–85.58% and 73.46–77.52% in grasses and sedges, respectively (Furch & Junk, 1997). The moisture content of freshwater macrophytes was lower than that of other Nigerian indigenous vegetables, such as *Amaranthus hybridus* (59.30%), *Telfaria occidentalis* (58.70%), and *B. alba* (54.80%) (Mepba, Eboh & Banigo, 2007).

The ash content, which is an index of mineral contents in biota, was high in *L. flava* (18.31%) compared to the values reported in young shoots and inflorescence *L. flava* (0.79%) (Saupi, Zakaria & Bujang, 2009). However, a similar value was observed for *M. balsamina* leaves (18.00%) (Hassan & Umar, 2006). In addition, the crude lipid content was also high in *L. flava* (5.75%), which was higher compared with the same species of *L. flava* at 1.22%, as reported by Saupi, Zakaria & Bujang (2009), as well as for *M. balsamina* leaves at 2.66% (Hassan & Umar, 2006). However, the content was lower than in some indigenous wild spices, herbs, fruits, and leafy vegetables, as reported by Achinewhu, Ogbonna & Hart (1995). The crude fibre content of *E. crassipes* was 21.34%, which was slightly lower than that of the green vegetables *M. balsamina* (29.00%) and *Myrianthus arboreus* (11.60%), both of which are consumed as soup in West Africa (Hassan & Umar, 2006).

The protein content in *N. oleracea* (29.61%) was comparable to that in *Moringa oleifera* (27.51%) (Oduro, Ellis & Owusu, 2008) and *Piper quineense* (26.6%) (Achinewhu, Ogbonna & Hart, 1995); additionally, it was higher than that in *M. arboreus* (18.75%) and *M. balsamina* leaves (11.29%) (Hassan & Umar, 2006). Higher protein in *N. oleracea* can be related to the excellent source of protein in the Leguminosae species. Plants of the legume family have root nodules in which symbiotic bacteria fix nitrogen to ammonia. This ammonia contains large amounts of proteins and amino acids (Roos et al., 2020). The crude protein content of edible *N. oleracea* was 46.37% of the recommended dietary allowance (RDA) (Institute of Medicine IOM, 2005). According to Adeyemi & Osubor (2016), *E. crassipes* contains higher nutritional values, especially in their leaf parts, which consist of concentrated forms of proteins. In addition, water hyacinth leaf protein contains many unsaturated fats, carotenes, xanthophylls, carbohydrates, and minerals, including calcium, iron, and phosphorus (Kateregga & Sterner, 2007). Hence, this wild vegetable can be considered a good protein supplement.

Based on the carbohydrate content, higher carbohydrate content was observed in *E. crassipes*, followed by *N. oleracea* and *L. flava*. In a study by Madsen, Luu & Getsinger (1993), water hyacinth roots were actively respiring tissues without any modifications for storing carbohydrates. Therefore, carbohydrates (such as sugar) accumulated in upper plant parts, such as leaf laminae, leaf petioles, and inflorescences, which can explain the higher carbohydrate content in *E. crassipes* compared to others. The carbohydrate content in all freshwater macrophytes (44.03–54.42%) of the current study was higher compared with *L. flava* young leaves and inflorescence (14.56%) that were examined by Saupi, Zakaria & Bujang (2009), as well as *M. balsamina* leaves (39.05%) examined by Hassan & Umar (2006) and some Nigerian edible leafy vegetables, such as *Basella alba* (34.30%), *Amaranthus hybridus* (27.40%), and *Lycopersicon esculentum* (33.20%), which were examined by Mepba, Eboh & Banigo (2007). Leafy vegetables are low lipid-containing foods; thus, they are beneficial in several health aspects, such as for avoiding obesity (Lintas, 1992). Vegetables usually contain low lipid content in a range of 0.10–0.20%, as reported by Hazra & Som (2005).

Fibre is one of the essential elements when consuming vegetables. Dietary fibre is part of an overall healthy diet to reduce blood cholesterol levels and to decrease heart disease risks and obesity. The fibre content was within the range of herbs (18.71–42.74%), as reported by Furch & Junk (1997). The benefit of consuming vegetables in human nutrition is represented by their high fibre content (Vadivel & Janardhanan, 2000). In addition, consuming large quantities of plant vegetables can provide adequate nutrients. *Eichhornia crassipes*, with a high fibre content, is similar to cellulosic wood and other lignocellulosic plants. They are also used as raw materials for papermaking (Wang et al., 2004; Saijonkari-Pahkala, 2001). A previous study by Banerjee & Matai (1990) reported that the leaf part normally had higher fibre content (especially in floating and emergent plants) because they required more strength to support the aerial vegetation. Fibre content is an important dietary component that is widely utilized as a value indicator in poultry and feeding animal diets. For food consumption, high fibre content helps to increase stool volume and decreases the time that waste products spend in the gastrointestinal tract (Enyi, Uwakwe & Wegwu, 2020). It has also been reported that a calorific value of more than 12% can be provided by plant food with a good source of crude protein (Pearson, 1976). Therefore, *N. oleracea* (29.61%), *L. flava* (16.58%), and other plants, such as *Alisma plantago-aquatica* (14.83%) and *N. nucifera* (14.05%), also meet this requirement.

Based on the National Diets and Nutrition Survey (NDNS) 2014, Bates et al. (2014) stated that the average percent carbohydrate uptake is 50% of the food consumed. Carbohydrates (starches) from cereals, roots, and tubers constitute primary energy-giving food, according to Achinewhu, Ogbonna & Hart (1995). Some indigenous leafy vegetables, nuts, and wild fruits constitute energy and can be provided as food supplements because they are also excellent carbohydrates. The energy value in *N. oleracea* (4,269.65 cal g^−1^) was shown to be high, comparable to that of the local vegetable *Ipomoea aquatica* (3,009.40 cal g^−1^) (Umar et al., 2007). In contrast, Umar et al. (2007) reported that most vegetables are low in energy value (within the range of 1,250–2,090 cal g^−1^). According to SACN (2011), the energy requirement for 19- to 75-year-old female adults is 1,840–2,175 kcal per day, whereas for 19- to 75-year-old male adults, the requirement is 2,272–2,294 kcal per day.

### Mineral content and comparative analysis with previous studies

**Figure 5:**
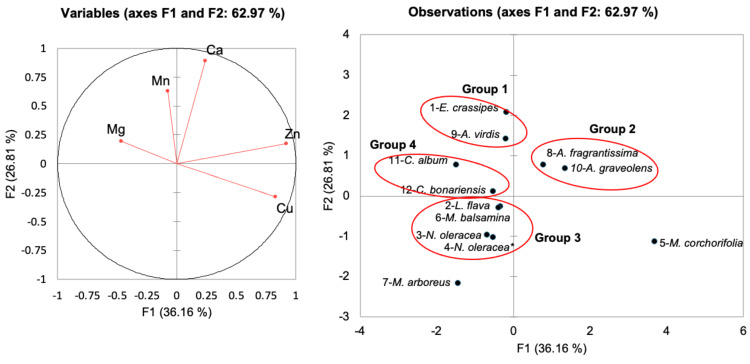
Principal component analysis of freshwater macrophytes with other edible plant species based on their mineral contents. (A) plot of mineral contents (B) position of PC score of species tested and other edible plants according to PC1 and PC2. No. 1–12 represents the assigned number of edible plant species as in Table 1 and corresponds to Groups 1, 2, 3, and 4.

Table 3 compares the mineral contents (macro- and micronutrients) of freshwater macrophyte species and other edible plants. The micronutrients (Cu, Mn, and Zn) in Table 4 were ordinated *via* a principal component analysis (PCA), as shown in Fig. 5. Some of the mineral elements were not included for comparison in the PCA, as no data from previous studies were available. The correlation matrix (Table 5) shows a significant moderate correlation between copper and zinc (*r* = 0.626).

**Table 3:** Comparison of mineral contents (macro- and micro-nutrient) of freshwater macrophyte species and other edible plant species.

**No.**	**Species**	**Macronutrients (mg kg** ^−1^ **)**					**Micronutrients (mg kg** ^−^ **1)**			
		**N**	**P**	**K**	**Ca**	**Mg**	**Cu**	**Mn**	**Zn**	**Fe**
**Freshwater macrophytes**										
1	*Eichhornia crassipes*	12380.0 ± 1129.9c (10040–16720)	657.0 ± 44.1b (488–824)	15799.0 ± 1708.9a (11716–27000)	11863.5 ± 316.4a (10764–13736)	1649.0 ± 24.7a (1560–1748)	27.0 ± 2.1a (20–36)	444.0 ± 18.7a (380–504)	158.5 ± 8.1a (120–196)	377.5 ± 31.8b (292-540)
2	*Limnocharis flava*	23970.0 ± 3022.0b (12960–35680)	2734.5 ± 417.1a (1964–5240)	33276.0 ± 10073.4a (17244–90400)	8066.5 ± 426.0b (6172–9284)	1198.5 ± 91.2b (980–1608)	16.0 ± 0.8b (12–20)	144.5 ± 20.8b (92–260)	110.0 ± 4.6b (92–128)	314.0 ± 25.4b (220-424)
3	*Neptunia oleracea*	40380.0 ± 730.6a (38320–41600)	2275.0 ± 232.7a (1924–2960)	11212.0 ± 94.3a (11008–11440)	3583.0 ± 120.2c (3416–3932)	2616.0 ± 68.0c (2488–2808)	20.0 ± 2.8^ab^ (16–28)	146.0 ± 14.3b (124–384)	107.0 ± 4.7b (100–120)	1282.0 ± 118.5a (984-1508)
	df	2	2	2	2	2	2	2	2	2
	F value	42.76	17.71	2.77	95.10	104.26	7.33	67.56	13.99	69.04
	*P* value	<0.0001	0.0005	0.1148	0.0001	0.0001	0.0110	0.0001	0.0013	0.0001
4	*Limnocharis flava* ^1^	–	–	42020	7708.7	2281	83.1	–	6.6	–
5	*Neptunia oleracea* ^2^	–	4059.2	32284	3814.2	1866.7	29.7	142.3	105.3	–
6	*Melochia corchorifolia* ^3^	–	1018.9	72.5	7503.7	1083.3	335	96.8	673	199.1
**Vegetables**										
7	*Momordica balsmina* ^4^	1224.9	1304.6	13200	9410	2200	54.4	116	31.8	–
8	*Amaranthus hybridus* ^5^	–	60	450	20	40	–	–	80	118
9	*Lycopersicon esculentum* ^5^	–	60	580	850	40	–	–	50	80
10	*Telfaria occidentalis* ^5^	–	40	280	50	360	–	–	130	800
11	*Basella alba* ^5^	–	60	–	10	60	–	–	20	60
12	*Myrianthus arboreus* ^6^	33800	5000	20130	540	4600	3.550	6.957	1.82	44.125
**Spices**										
13	*Piper quineense* ^7^	10800	300	700	11500	3500	–	–	–	-
**Medicinal plants**										
14	*Fritillaria ussuriensis* ^8^	–	–	–	355.76	–	3.44	12.91	31.54	103.88
15	*Gastrodia elata* ^8^	–	–	–	1415.34	–	3.42	30.09	10.64	126.15
16	*Achillea fragrantissima* ^9^	–	–	–	14400	1200	4.44	88.5	400	192
17	*Amaranthus virdis* ^9^	–	–	–	15280	8255	12.44	108.1	356	480
18	*Asteriscus graveolens* ^9^	–	–	–	13200	1050	26.8	107.1	544	204
19	*Chenopodium album* ^9^	–	–	–	11500	8900	14.44	148	21.2	380
20	*Conyza bonariensis* ^9^	–	–	–	10200	1092	8.2	152.6	38.8	255.1

**Notes.**

The varying superscript alphabets in the same column demonstrate the contrasts at *p* < 0.05 (ANOVA, Duncan’s Multiple Range Test test). Values are given as mean ± SE and range in parenthesis. 1–9 are references list; 1&2- Saupi, Zakaria & Bujang (2009); 3- Umar et al. (2007); 4- Hassan & Umar (2006); 5- Mepba, Eboh & Banigo (2007); 6- Amata (2010); 7- Achinewhu, Ogbonna & Hart (1995); 8-Yukui et al. (2016); 9- Daur (2015).

**Table 4:** Comparison of mineral contents of freshwater macrophyte species and other edible plant species.

**No**	**Species**	**Ca**	**Mg**	**Cu**	**Mn**	**Zn**	**Reference (s)**
		**(mg kg** ^−1^ **)**					
1	*Eichhornia crassipes*	11,863.5	1,649	27.0	444.0	158.5	Present study
2	*Limnocharis flava*	8,066.5	1,198.5	16	144.5	110.0	Present study
3	*Neptunia oleracea*	3,583	2,616	20	146	107	Present study
4	*Neptunia oleracea* ^*^	3,814.2	1,866.7	29.7	142.3	105.3	Saupi, Zakaria & Bujang (2009)
5	*Melochia corchorifolia*	7,503.7	1,083.3	335	96.8	673	Umar et al. (2007)
6	*Momordica balsamina*	9,410	2,200	54.4	116	31.8	Hassan & Umar (2006)
7	*Myrianthus arboreus*	540	4,600	3.55	6.957	1.82	Amata (2010)
8	*Achillea fragrantissima*	14,400	1,200	4.44	88.5	400	Daur (2015)
9	*Amaranthus virdis*	15,280	8,255	12.44	108.1	356	Daur (2015)
10	*Asteriscus graveolens*	13,200	1,050	26.8	107.1	544	Daur (2015)
11	*Chenopodium album*	11,500	8,900	14.44	148	21.2	Daur (2015)
12	*Conyza bonariensis*	10,200	1,092	8.2	152.6	38.8	Daur (2015)

**Notes.**

* *Neptunia oleracea* study by Saupi, Zakaria & Bujang (2009).

Minerals are essential in the diet, even at contents of 4–6% of the human body. The daily body (per day) macro mineral requirement is higher than 100 mg. In addition, they serve as structural components of tissues and for functional cellular and basal metabolism (Macrae, Robinson & Sadler, 1993). The contents of N, P, Mg, Ca, Cu, Mn, Fe, and Zn were found to be different among the three plant species studied(except for K). The protein and mineral contents of the water in which the plants are grown are strongly reliant on the composition of the water; for example, the protein and phosphorus contents of the plant are directly proportional to the nutrient loading rate of the water (Boyd, 1970). As stated above, higher ash was observed in *L. flava* (18.31%) and *E. crassipes* (13.23%), which contributed to their higher mineral composition. From the current study, nitrogen values ranged from 12,380–40,380 mg kg^−1^. They were higher than those of *M. balsmina* (1,224.9 mg kg^−1^) and *P. quineense* (10,800 mg kg^−1^) but comparable to those of *M. arboreus* (33,800 mg kg^−1^), which showed that freshwater macrophytes are nitrogen-rich vegetables. Moreover, the phosphorus and potassium contents of *L. flava* (2,734.5 mg kg^−1^ and 33,276.0 mg kg^−1^, respectively) were higher than those of *Melochia corchorifolia* (1,018.9 and 72.5 mg kg^−1^, respectively), *M. balsmina* (1,304.6 and 13,200 mg kg^−1^, respectively), and *P. quineense* (300 and 700 mg kg^−1^, respectively). However, its *P* value was lower than that of freshwater *N. oleracea* (4,059.2 mg kg^−1^) and the vegetable *M. arboreus* (5,000 mg kg^−1^) from previous studies by Saupi, Zakaria & Bujang (2009) and Hassan & Umar (2006). Additionally, the calcium content in *L. flava* was higher (8,066.5 mg kg^−1^) than that in the same species in prior studies (7,708.7 mg kg^−1^), as well as other freshwater macrophyte species, including *N. oleracea* (3,814.2 mg kg^−1^) and *M. corchorifolia* (7,503.7 mg kg^−1^), and most of the vegetables (except for *M. balsmina*) (9,410 mg kg^−1^). The Mg values of the current study ranged from 1,198.5 to 2,716.0 mg kg^−1^. Furthermore, they were comparable with freshwater macrophytes from previous studies (range between 1,083–2,281 mg kg^−1^) and vegetables of *M*. *balsmina* and some medicinal plants, such as *A. fragrantissima*, *A. graveolens,* and *C. bonariensis* (1,200, 1,050, and 1,092 mg kg^−1^, respectively).

**Table 5:** Correlation matrix for Ca, Mg, Cu, Mn, and Zn in freshwater macrophyte species with other edible plant species.

**Variables**	**Ca**	**Mg**	**Cu**	**Mn**	**Zn**
Ca	1.000	0.157	−0.110	0.271	0.413
Mg		1.000	−0.246	−0.164	−0.242
Cu			1.000	−0.096	0.626^*^
Mn				1.000	−0.133
Zn					1.000

**Notes.**

* significant at *p* < 0.05.

Sodium, potassium, phosphorus, and magnesium are macronutrients that play an essential role in calcium homeostasis and bone status (Heaney, 2015). Furthermore, fruits and vegetables that are rich in potassium have an alkaline ash characteristic that is important in the diet. Adequate phosphorus indicates sufficient protein content in a healthy diet. Both calcium and phosphorus play a role in the growth and maintenance of muscles, bones, and teeth (Turan et al., 2003). According to the National Coordinating Committee on Food and Nutrition (NCCFN) Ministry of Health Malaysia(2017), the calcium intake for men and women aged 19–29-years-old was 800 mg, whereas for pregnant women, this intake was was 1,000 mg. Therefore, the value of calcium in this plant indicates that this plant may serve as a vital calcium source.

Based on the Bray–Curtis similarity index at 50% similarity, Fig. 5 shows that the total variance of the first two components was 62.97% (PC1 had a total variance of 36.16%, and PC2 had a variance of 26.81%). *Eichornia crassipes* was grouped with a medicinal plant (*A. virdis*) in Group 1 due to higher Mn (444.0 and 108.1 mg kg^−1^, respectively) and similar Cu values (27.0 mg kg^−1^ for *E. crassipes* and 26.8 mg kg^−1^ for *A. graveolens*). Group 2 consisted of medicinal plants, including *A. fragrantissima,* and *A. graveolens,* with higher Zn contents (400 and 544 mg kg^−1^, respectively). The recommended zinc uptake for men and women (19–29-years-old) is 6.70 mg and 4.90 mg, respectively, whereas for pregnant women, this intake is 5.50–10.00 mg (National Coordinating Committee on Food and Nutrition, NCCFN) Ministry of Health Malaysia(2017). Zinc deficiency results in retarded growth and delayed sexual maturation (Berminas, Charles & Emmanuel, 1998). Both *L*. *flava* and *N*. *oleracea* were clustered in Group 3 with *N. oleracea* and *M. balsmina.* Moreover, *C. album* and C. *bonariensis* were clustered in Group 4, with comparable Mg values ranging from 1,092–8,900 to 196 mg kg^−1^.

Furthermore, iron (Fe) is one of the essential micronutrients in forming haemoglobin and for the functioning of the central nervous system in the body (Adeyeye & Otokiti, 1999). The results showed that *L. flava* and *E. crassipes* exhibited iron levels at 314.0 mg kg^−1^ and 377.5 mg kg^−1^, respectively, compared with the iron values in medicinal plants (*C. bonariensis* and C. *album*), which ranged from 255.1–380 mg kg^−1^. The high iron content in the plant could be the reason as to why iron requirements are higher in pregnancy than in the nonpregnant state.

## Conclusions

Although they are thought to be aquatic weeds due to their fast growth and nuisance to the aquatic environment, freshwater macrophytes are also consumed as food, especially by the local residents, for their subsistence. The results of the current study showed that the species had higher and comparable protein (*L. flava* and *N. oleracea*) and carbohydrate (*E. crassipes* and *N. oleracea*) contents than other edible vegetables that are suitable for human consumption as energy sources. An adequate amount of ash (*L. flava* and *E. crassipes*) and mineral analysis in each plant species can serve as alternatives in food nutrient supplements. These aquatic plants are also useful staple foods because they are simple to cultivate, spread quickly, and require minimal care.

##  Supplemental Information

10.7717/peerj.15496/supp-1 Supplemental Information 1 Raw data of macronutrient and micronutrient contents and principal component analysis of freshwater macrophytes with other edible plant species based on their mineral contents

10.7717/peerj.15496/supp-2 Supplemental Information 2 Raw data of proximate contents and principal component analysis of freshwater macrophytes with other edible plant species based on their proximate composition

10.7717/peerj.15496/supp-3 Supplemental Information 3 Raw data of principal component analysis of freshwater macrophytes with other edible plant species based on their mineral contents

## Additional Information and Declarations

### Data Availability

The following information was supplied regarding data availability: The raw data is in the Supplementary Files.

## References

[ref-1] Association of Official Agricultural Chemists (AOAC) (2000). 17th Edition, The Association of Official Analytical Chemists, Gaithersburg, MD, USA. Methods 925.10, 65.17, 974.24, 992.16.

[ref-2] Abubakar S, Afolayan M, Osuji C, Sallau A, Alabi O (2021). A comparative study of physicochemical, proximate and minerals analysis of some underutilized wild edible seeds used as condiments in Nigerian traditional soups. World Journal of Biology Pharmacy and Health Sciences.

[ref-3] Achinewhu SC, Ogbonna CC, Hart AD (1995). Chemical composition of indigenous wild herbs, spices, fruits, nuts and leafy vegetables used as food. Plants Food for Human Nutrition.

[ref-4] Addinsoft SA (2015). https://www.xlstat.com/en/.

[ref-5] Adeyemi O, Osubor CC (2016). Assessment of nutritional quality of water hyacinth leaf protein concentrate. The Egyptian Journal of Aquatic Research.

[ref-6] Adeyeye EI, Otokiti MKO (1999). Proximate composition and some nutritional valuable minerals of two varieties of *Capsicum annum* (Bells and Cherry Peppers). International Journal of Engineering, Science and Technology.

[ref-7] Almatsier ZS (2006). Prinsip Dasar Ilmu Gizi.

[ref-8] Amata IA (2010). Nutritive value of the leaves of *Myrianthus arboreus*: a browse plant. International Journal of Agricultural Research.

[ref-9] Bates B, Lennox A, Prentice A, Bates C, Page P, Nicholson S, G Swan (2014). National Diet and Nutrition Survey (NDNS) results from years 1, 2, 3 and 4 (combined) of the rolling programmed (2008/2009–2011/2012).

[ref-10] Banerjee A, Matai S (1990). Composition of Indian aquatic plants in relation to utilization as animal forage. The Journal of Aquatic Plant Management.

[ref-11] Berminas JT, Charles M, Emmanuel D (1998). Mineral composition of non-conventional leafy vegetables. Plant Food for Human Nutrition.

[ref-12] Boyd CE (1970). Vascular aquatic plants for mineral nutrient removal from polluted waters. Economic Botany.

[ref-13] Caunii A, Cuciureanu R, Zakar AM, Tonea E, Giuchici C (2010). Chemical composition of common leafy vegetables. Studia Universitatis Vasile Goldis, Seria Stiintele Vietii.

[ref-14] Dastagir G, Hussain F, Khattak F, Khandzadi O (2013). Proximate analysis of plants of family Zygophyllaceae and Euphorbiaceae during winter. Sarhad Journal of Agriculture.

[ref-15] Daur I (2015). Chemical composition of selected Saudi medicinal plants. Arabian Journal of Chemistry.

[ref-16] Den Hartog C (1981). Aquatic plant communities of Poikilosaline waters. Hydrobiologia.

[ref-17] Enyi CN, Uwakwe AA, Wegwu MO (2020). Nutritional potentials of water hyacinth (*Eichhornia crassipes*). Direct Research Journal of Public Health and Environmental Technology.

[ref-18] Ezzati M, Lopez AD, Rodgers A, Vander HS, Christopher JLM (2002). Selected major risk factors and global and regional burden of disease. The Lancet.

[ref-19] Furch K, Junk WJ, Furch K, Junk WJ (1997). The chemical composition, food value, and decomposition of herbaceous plants, leaves, and leaf litter of floodplain forests. The central amazon floodplain: ecology of a pulsing system.

[ref-20] Ghanimi R, Ouhammou A, Baazizi H, Houguig K, Cherkaoui M (2022). Nutritional value of six wild edible plants traditionally used as vegetables in Morocco. Ecology, Environment and Conservation.

[ref-21] Grubben GJH, Siemonsma JS, Kasem P, Siemonsma JS, Kasem P (1994). Introduction. Plant resources of South-East Asia No. 8: vegetables.

[ref-22] Gupta S, Bains K (2006). Traditional cooked vegetable dishes as important sources of ascorbic acid and *β*-carotene in the diets of Indian urban and rural families. Food and Nutrition Bulletin.

[ref-23] Hassan LG, Umar KJ (2006). Nutritional value of balsam apple (*Momordica balsamina* L.) leaves. Pakistan Journal of Nutrition.

[ref-24] Hazra P, Som MG (2005). Vegetable science.

[ref-25] Heaney RP, Holick M, Nieves J (2015). Sodium, potassium, phosphorus and magnesium. Nutrition and bone health. Nutrition and health.

[ref-26] Igwenyi IO, Offor CE, Ajah DA, Nwankwo OC, Ukaomah JI, Aja PM (2011). Chemical compositions of *Ipomoea aquatica* (green kangkong). International Journal of Pharma and Bio Sciences.

[ref-27] Institute of Medicine (IOM) (2005). Dietary reference intakes for nergy, carbohydrate, fiber, fat, fatty acids, cholesterol, protein, and amino acids (Macronutrients).

[ref-28] Kateregga E, Sterner T (2007). Indicators for an invasive species: water hyacinths in Lake Victoria. Ecological Indicators.

[ref-29] Lacoul P, Freedman B (2006). Environmental influences on aquatic plants in freshwater ecosystems. Environmental Reviews.

[ref-30] Lintas C, Lauret F (1992). Nutritional aspects of fruits and vegetable consumption. Les fruits et légumes dans les économies méditerranéennes: actes du colloque de Chania.

[ref-31] Macrae R, Robinson RK, Sadler MJ (1993). Encyclopaedia of food science, food technology and nutrition.

[ref-32] Madsen JD, Luu KT, Getsinger KD (1993). Allocation of Biomass and Carbohydrates in Waterhyacinth (Eichhornia crassipes): Pond-Scale Verification. Technical Report A-93-3. Vicksburg: U.S. Army Engineer Waterways Experiment Station. Options Méditerranéennes: Série A. Séminaires Méditerranéens. Colloque sur les Fruits et Légumes dans les Economies Méditerranéennes.

[ref-33] Mepba HD, Eboh L, Banigo DEB (2007). Effects of processing treatments on the nutritive composition and consumer acceptance of some Nigerian edible leafy vegetables. African Journal of Agricultural Nutrition and Development.

[ref-34] Mertz W (1982). Trace minerals and atherosclerosis. Federation Proceedings.

[ref-35] Muta Harah Z, Japar Sidik B, Suzalina Akma A, Nurul Amin SM, Mohd Salleh K, Aziz A, Romano N (2014). Aquatic macrophytes and their usefulness. Perspective of fisheries and aquaculture in Malaysia.

[ref-36] Muta Harah Z, Japar Sidik B, Raesah A, Maini C, Suzalina AA (2005). Aquatic macrophytes in natural and manmade water bodies. Bio-Science Research Bulletin.

[ref-37] National Coordinating Committee on Food and Nutrition (NCCFN) Ministry of Health Malaysia (2017). Recommended Nutrient Intakes for Malaysia (RNI). A report of the technical working group on nutritional guidelines.

[ref-38] Noorasmah S, Muta Harah Z, Japar Sidik B, Arshad A (2016). Growth performance and production of *Limnocharis flava* (l.) Buchenau for vegetable crop. International Journal of Agriculture and Environmental Research.

[ref-39] Noorasmah S, Muta Harah Z, Sidik BJapar, Arshad A (2015). The proximate compositions and mineral contents of *Neptunia oleracea* Loureiro, an aquatic plant from Malaysia. Emirates Journal of Food and Agriculture.

[ref-40] Oduro I, Ellis WO, Owusu D (2008). Nutritional potential of two leafy vegetables: *Moringa oleifera* and *Ipomoea batatas* leaves. Scientific Research and Essay.

[ref-41] Pearson D (1976). Chemical analysis of foods.

[ref-42] Roos E, Carlsson G, Ferawati F, Hefni M, Stephan A, Tidaker P, Witthoft C (2020). Less meat, more legumes: prospects and challenges in the transition toward sustainable diets in Sweden. Renewable Agriculture and Food Systems.

[ref-43] Saijonkari-Pahkala K (2001). Non-wood plants as raw material for pulp and paper. Agricultural and Food Science in Finland. Academic Dissertation.

[ref-44] Saupi N, Saidin AA, Zakaria MH, Sarbini SR, Yusli NA (2020). An ethnobotanical study of indigenous leafy vegetables among local communities in Bintulu, Sarawak, Malaysia. Borneo Journal of Resource Science and Technology.

[ref-45] Saupi N, Zakaria MH, Bujang JS (2009). Analytic chemical composition and mineral content of yellow velvetleaf (*Limnocharis flava* L. Buchenau)’s edible parts. Journal of Applied Sciences.

[ref-46] Scientific Advisory Committee on Nutrition (SACN) (2011). Dietary reference values for energy.

[ref-47] Seal T, Pillai B, Chaudhuri K (2017). Nutritional potential of five unexplored wild edible plants consumed by the tribal people of Arunachal Pradesh state in India. International Journal of Food Science and Nutrition.

[ref-48] Shad AA, Shah HU, Bakht J (2013). Ethnobotanical assessment and nutritive potential of wild food plants. Journal of Animal and Plant Sciences.

[ref-49] Soerjani M, Kostermans AJ, Tjitrosoepomo G (1987). Weeds of rice in Indonesia.

[ref-50] Stratton AE, Finley JW, Gustafson DI, Mitcham EJ, Myers SS, Naylor RL, Otten JJ, Palm CA (2021). Mitigating sustainability tradeoffs as global fruit and vegetable systems expand to meet dietary recommendations. Environmental Research Letters.

[ref-51] Tbatou M, Kabil M, Belahyan A, Belahsen R (2018). Dietary potential of some forgotten wild leafy vegetables from Morocco. International Food Research Journal.

[ref-52] Turan MS, Kordali H, Zenglin H, Dursan A, Sezen Y (2003). Macro and micro mineral content of some wild edible leaves consumed in Eastern Anatolia. Acta Agriculturae Scandinavica, Section B—Plant Soil Science.

[ref-53] Umar KJ, Hassan LG, Dangoggo SM, Inuwa M, Amustapha MN (2007). Nutritional content of *Melochia corchorifolia* (Linn.) leaves. International Journal of Biological Chemistry.

[ref-54] Vadivel V, Janardhanan K (2000). Chemical composition of the underutilized legume *Cassia hirsuta* L. Plant Foods for Human Nutrition.

[ref-55] Wang S, Cheng Q, Rials TG, Lee SH (2004). Cellulose Microfibril/Nanofibril and its Nanocomposites.

[ref-56] Yukui R, Xiaofei C, Ying Y, Rui G (2016). Contents of mineral elements in two traditional Tibetan medicines *Fritillaria ussuriensis* and *Gastrodia elata*. Arabian Journal of Chemistry.

[ref-57] Zar JH (1999). Biostatistic analysis.

